# Supplementary material for: A Likelihood Approach for Real-Time Calibration of Stochastic Compartmental Epidemic Models
Source: PLoS Comput Biol. 2017 Jan 17;13(1):e1005257. doi: 10.1371/journal.pcbi.1005257 (PMC5240920; doi:10.1371/journal.pcbi.1005257)
Supplement: S1 File — (TAR.GZ) [file pcbi.1005257.s014.tar.gz › HSPH_Online-SI-Revision/PF08/n10-severe/n10-severe_PF_table-8.pdf]

1	R0	{1.68952, 11.8402}	{1.63139, 18.6543}	{{1.48308, 14.1795}, {1.74557, 20.}}	{7.2075, 57.5506}	inf	217.	513.437	{391.619, 660.016}	136.607	0.365029	newly	155.	209.33	{153., 273.}	36.7806	0.129004	Reff	1.60166	1.44487	{1.326, 1.58458}	10.5269	ar	6872.	7482.85	{6682., 8243.}	10.0811
2	R0	{1.71739, 9.62661}	{1.69048, 18.6341}	{{1.48151, 13.9967}, {2.0355, 20.}}	{11.1733, 93.5685}	inf	89.	407.244	{288.535, 537.794}	357.578	0.648232	newly	86.	168.92	{115., 225.}	96.4186	0.278629	Reff	1.66844	1.53494	{1.35532, 1.78649}	11.6315	ar	6874.	7441.97	{6371., 8739.}	11.5471
3	R0	{1.48936, 6.23975}	{1.75024, 16.9744}	{{1.37783, 11.8119}, {2.30234, 20.}}	{21.4053, 172.037}	inf	75.	316.998	{170.296, 526.926}	322.665	0.587519	newly	102.	155.34	{74., 270.}	64.0588	0.196193	Reff	1.45406	1.63176	{1.29718, 2.09693}	18.9012	ar	5666.	7196.32	{5504., 8986.}	28.6971
4	R0	{1.62394, 7.51135}	{1.5618, 19.4465}	{{1.50038, 18.6099}, {1.63425, 20.}}	{4.90563, 158.895}	inf	51.	488.209	{419.447, 563.318}	857.273	0.97616	newly	60.	186.69	{150., 220.}	211.15	0.487108	Reff	1.59877	1.37591	{1.32128, 1.45648}	13.9391	ar	6417.	7113.63	{6639., 7577.}	11.0655
5	R0	{1.53632, 8.57652}	{1.59249, 19.5375}	{{1.4829, 18.7108}, {1.69408, 20.}}	{5.19371, 127.803}	inf	91.	551.426	{458.739, 684.564}	505.962	0.776529	newly	100.	212.72	{173., 266.}	112.72	0.320694	Reff	1.48962	1.4052	{1.3454, 1.4956}	6.72823	ar	6260.	7382.27	{6854., 8042.}	17.9276
6	R0	{1.47166, 5.7434}	{1.61032, 19.4143}	{{1.52144, 18.7124}, {1.72865, 20.}}	{9.71169, 238.029}	inf	45.	467.279	{376.672, 565.71}	938.398	1.00849	newly	63.	182.92	{141., 219.}	190.349	0.453769	Reff	1.45297	1.45393	{1.36943, 1.56825}	4.16887	ar	5650.	7361.7	{6761., 8002.}	30.2956
7	R0	{1.48179, 8.87369}	{1.64616, 19.2902}	{{1.4739, 18.3763}, {1.87686, 20.}}	{11.4364, 117.386}	inf	250.	613.43	{448.742, 846.314}	145.372	0.377636	newly	229.	239.93	{166., 319.}	22.1266	0.0933445	Reff	1.37169	1.4251	{1.3184, 1.55743}	6.08473	ar	5233.	7613.27	{6776., 8578.}	45.4858
8	R0	{1.53669, 6.47807}	{1.69986, 18.423}	{{1.45126, 13.8072}, {2.065, 20.}}	{13.0897, 184.39}	inf	77.	345.95	{243.558, 488.445}	349.285	0.637837	newly	107.	147.99	{100., 197.}	41.243	0.138672	Reff	1.50611	1.58022	{1.36635, 1.8976}	10.6538	ar	5934.	7428.76	{6335., 8724.}	25.4193
9	R0	{1.57227, 6.20989}	{1.96333, 16.2074}	{{1.24963, 11.0173}, {2.79456, 20.}}	{32.826, 160.993}	inf	9.	233.163	{80.0459, 386.475}	2490.7	1.32759	newly	13.	126.19	{36., 250.}	870.692	0.885795	Reff	1.56598	1.84938	{1.19378, 2.47322}	28.0077	ar	6136.	7388.9	{4151., 9452.}	33.057
10	R0	{1.54936, 6.50938}	{1.92422, 15.7012}	{{1.24644, 9.50898}, {2.53006, 20.}}	{33.3553, 141.48}	inf	30.	193.655	{62.3011, 366.239}	545.516	0.720865	newly	40.	110.16	{33., 194.}	182.95	0.379714	Reff	1.53789	1.8291	{1.20407, 2.4484}	29.9028	ar	6275.	7200.89	{4185., 9285.}	31.2368
11	R0	{1.42179, 3.18601}	{1.72782, 15.3105}	{{1.2447, 9.47618}, {2.44165, 20.}}	{28.4348, 380.555}	inf	3.	207.374	{85.4219, 404.157}	6812.47	1.76793	newly	12.	113.82	{44., 200.}	848.5	0.8998	Reff	1.40601	1.62978	{1.16058, 2.3608}	26.0095	ar	5322.	6483.76	{4054., 9033.}	34.3397
12	R0	{1.48035, 6.46183}	{1.86814, 17.1875}	{{1.38522, 12.3252}, {2.35251, 20.}}	{28.9447, 165.984}	inf	26.	256.389	{132.278, 442.293}	886.11	0.950472	newly	35.	124.28	{56., 211.}	255.086	0.503573	Reff	1.47147	1.77802	{1.32961, 2.22771}	24.9097	ar	5793.	7648.99	{5432., 9184.}	34.3223
13	R0	{1.75755, 8.53854}	{1.73248, 18.9748}	{{1.47914, 16.1294}, {2.08353, 20.}}	{11.2932, 122.225}	inf	33.	358.951	{245.869, 503.341}	987.73	1.01683	newly	37.	149.85	{95., 229.}	305.	0.582778	Reff	1.74472	1.61623	{1.38693, 1.87099}	10.9883	ar	6832.	7608.98	{6486., 8756.}	13.8879
14	R0	{1.47355, 4.89451}	{1.77588, 17.3932}	{{1.4111, 12.8606}, {2.29958, 20.}}	{22.4265, 255.361}	inf	20.	276.22	{161.117, 435.614}	1281.1	1.10385	newly	45.	133.84	{65., 257.}	197.422	0.419742	Reff	1.4625	1.67355	{1.33081, 2.19483}	18.578	ar	5710.	7321.88	{5567., 9050.}	29.6245
15	R0	{1.62071, 5.98665}	{1.9435, 13.1068}	{{1.14096, 6.10929}, {2.86095, 19.0269}}	{33.7794, 125.026}	inf	11.	136.527	{7.99546, 313.302}	1154.89	0.939397	newly	20.	113.14	{7., 206.}	487.9	0.678403	Reff	1.61763	1.85126	{1.091, 2.71041}	30.8445	ar	6635.	6605.44	{1756., 9257.}	32.1227
16	R0	{1.67268, 7.7256}	{1.80179, 17.1319}	{{1.37244, 11.0586}, {2.46091, 20.}}	{18.4362, 121.755}	inf	103.	295.209	{160.532, 495.74}	186.659	0.412424	newly	121.	143.03	{68., 226.}	44.0579	0.168513	Reff	1.63555	1.69053	{1.3023, 2.1669}	16.4758	ar	6947.	7417.59	{5383., 9186.}	17.9345
17	R0	{1.67805, 11.1417}	{1.73121, 16.4584}	{{1.36582, 11.7671}, {2.21235, 20.}}	{17.0628, 50.1985}	inf	109.	262.909	{147.626, 418.693}	141.201	0.34391	newly	87.	131.9	{69., 222.}	59.8621	0.181047	Reff	1.646	1.63191	{1.28749, 2.10175}	16.0722	ar	6625.	7094.38	{5144., 8876.}	19.242
18	R0	{1.66743, 9.99046}	{1.78924, 18.3505}	{{1.52314, 13.5871}, {2.14006, 20.}}	{13.6938, 83.6803}	inf	118.	415.934	{268.5, 666.76}	252.487	0.521143	newly	114.	180.2	{114., 284.}	60.1579	0.180921	Reff	1.61574	1.64107	{1.40021, 1.9187}	11.5609	ar	6899.	7789.95	{6710., 8963.}	15.0983
19	R0	{1.70787, 9.85478}	{1.59497, 19.483}	{{1.51025, 18.6587}, {1.65311, 20.}}	{7.72006, 97.7009}	inf	143.	532.944	{429.981, 649.922}	272.688	0.564526	newly	120.	203.97	{157., 246.}	69.975	0.22273	Reff	1.63409	1.39032	{1.33266, 1.46662}	14.9181	ar	6867.	7288.92	{6825., 7781.}	6.76249
20	R0	{1.53809, 10.5848}	{1.58959, 19.4779}	{{1.49036, 18.0563}, {1.67558, 20.}}	{4.69814, 84.0178}	inf	127.	540.458	{457.67, 634.298}	325.558	0.624691	newly	110.	206.39	{168., 251.}	87.6273	0.268135	Reff	1.48364	1.40001	{1.32731, 1.48305}	6.0714	ar	6108.	7391.32	{6836., 7805.}	21.0105
21	R0	{1.59925, 6.74479}	{2.00096, 15.7662}	{{1.22062, 10.0331}, {2.89293, 20.}}	{35.5538, 134.1}	inf	35.	218.808	{74.5251, 377.23}	525.164	0.703954	newly	45.	128.66	{34., 265.}	195.511	0.395058	Reff	1.58662	1.89337	{1.17399, 2.6208}	32.0239	ar	6411.	7384.13	{3837., 9488.}	31.4683
22	R0	{1.38779, 4.40977}	{1.92447, 17.3919}	{{1.39895, 12.5615}, {2.6148, 20.}}	{39.2556, 294.395}	inf	43.	324.035	{149.475, 577.826}	653.57	0.821658	newly	75.	155.42	{64., 272.}	112.88	0.285046	Reff	1.36101	1.80195	{1.33447, 2.44245}	33.6996	ar	5154.	7718.56	{5540., 9503.}	50.3574
23	R0	{1.53657, 7.50352}	{1.73945, 17.9125}	{{1.39914, 13.6124}, {2.15205, 20.}}	{17.6996, 138.721}	inf	100.	320.311	{179.213, 486.351}	220.311	0.474244	newly	116.	142.7	{78., 222.}	41.5172	0.150981	Reff	1.50353	1.61654	{1.32033, 1.93097}	14.3801	ar	5928.	7259.01	{5599., 8638.}	25.1678
24	R0	{1.71131, 6.24526}	{1.78863, 17.789}	{{1.41191, 13.3582}, {2.18008, 20.}}	{15.4254, 184.84}	inf	12.	279.752	{169.118, 419.491}	2231.26	1.33491	newly	18.	129.55	{72., 203.}	619.722	0.818047	Reff	1.70104	1.67854	{1.35233, 2.0762}	14.0249	ar	6924.	7447.67	{5683., 8829.}	16.1313
25	R0	{1.4412, 3.07729}	{2.02089, 14.6767}	{{1.35324, 9.79679}, {2.80242, 20.}}	{42.9301, 376.935}	inf	5.	201.963	{77.5891, 366.792}	3939.26	1.5369	newly	20.	134.53	{41., 256.}	572.65	0.726546	Reff	1.43789	1.92766	{1.29143, 2.62844}	38.0917	ar	5810.	7664.62	{4998., 9478.}	38.3057
26	R0	{1.4159, 4.93858}	{1.99714, 16.2144}	{{1.2265, 10.7139}, {2.79459, 20.}}	{45.3746, 228.321}	inf	18.	212.525	{75.9084, 368.135}	1080.69	0.990551	newly	30.	120.41	{32., 216.}	304.967	0.513255	Reff	1.40925	1.89582	{1.17399, 2.52694}	40.4973	ar	5255.	7439.12	{3926., 9330.}	50.3402
27	R0	{1.57465, 7.05414}	{2.13745, 14.7403}	{{1.37855, 9.78928}, {3., 20.}}	{41.3975, 109.603}	inf	15.	196.887	{84.3371, 343.348}	1212.58	1.05537	newly	22.	140.19	{38., 275.}	538.5	0.696984	Reff	1.57134	2.04037	{1.30473, 2.87103}	37.4267	ar	6359.	7839.16	{5172., 9597.}	32.4444
28	R0	{1.41422, 4.62402}	{1.98738, 16.541}	{{1.24445, 10.7139}, {2.98705, 20.}}	{44.4999, 257.719}	inf	6.	204.788	{67.3541, 365.793}	3313.13	1.44226	newly	14.	111.87	{31., 191.}	699.643	0.791003	Reff	1.41153	1.89704	{1.19246, 2.72565}	39.8364	ar	5485.	7385.42	{4050., 9593.}	44.0091
29	R0	{1.41901, 3.63017}	{1.99493, 13.4509}	{{1.1546, 6.93514}, {2.86001, 18.8819}}	{46.6476, 271.171}	inf	3.	142.698	{8.16113, 315.841}	4657.53	1.42127	newly	4.	99.18	{7., 206.}	2385.5	1.1883	Reff	1.4183	1.89753	{1.14204, 2.64568}	41.3773	ar	5979.	6973.24	{2558., 9257.}	38.3225
30	R0	{1.60719, 4.72984}	{2.00543, 13.5521}	{{1.1546, 8.23415}, {2.86001, 18.8819}}	{35.3102, 187.982}	inf	1.	145.659	{8.16113, 315.841}	14 465.9	1.90883	newly	2.	101.	{7., 207.}	4954.	1.4735	Reff	1.60687	1.90754	{1.14204, 2.64568}	31.1456	ar	6479.	7019.93	{2558., 9257.}	31.7977
31	R0	{1.38175, 6.26468}	{1.86333, 16.4532}	{{1.25128, 11.2301}, {2.45544, 20.}}	{38.123, 162.842}	inf	64.	257.483	{87.9426, 464.687}	302.521	0.523846	newly	96.	131.12	{40., 263.}	68.6458	0.241468	Reff	1.35577	1.74838	{1.19904, 2.34976}	33.9823	ar	5397.	7071.22	{4164., 9119.}	40.1338
32	R0	{1.4947, 8.8847}	{1.6342, 19.4903}	{{1.49909, 18.8866}, {1.8144, 20.}}	{9.4609, 119.369}	inf	208.	610.069	{460.269, 754.23}	193.302	0.456543	newly	186.	237.25	{179., 307.}	29.4785	0.103555	Reff	1.38021	1.42767	{1.35848, 1.51422}	4.60555	ar	5407.	7604.34	{6965., 8263.}	40.6388
33	R0	{1.55891, 8.28978}	{1.54859, 19.3965}	{{1.45188, 18.7805}, {1.64724, 20.}}	{4.27316, 133.981}	inf	149.	516.865	{420.65, 664.378}	246.889	0.534249	newly	142.	199.12	{161., 250.}	40.4507	0.140632	Reff	1.49203	1.37862	{1.3128, 1.46181}	7.96959	ar	6239.	7176.4	{6655., 7681.}	15.0248
34	R0	{1.60764, 10.4729}	{1.60037, 19.5685}	{{1.48411, 18.9223}, {1.6873, 20.}}	{4.54158, 86.8494}	inf	256.	578.456	{450.858, 699.907}	125.96	0.34682	newly	188.	222.48	{170., 264.}	22.2872	0.0829403	Reff	1.49125	1.41542	{1.33695, 1.51489}	6.07032	ar	6495.	7482.97	{6912., 7990.}	15.2112
35	R0	{1.48527, 5.74485}	{1.93943, 17.1801}	{{1.44095, 10.5552}, {2.58226, 20.}}	{31.9628, 199.053}	inf	25.	276.499	{138.626, 504.505}	1005.99	0.990194	newly	43.	140.26	{56., 250.}	226.419	0.445989	Reff	1.47606	1.83831	{1.38842, 2.46129}	27.2631	ar	5811.	7815.51	{5984., 9436.}	35.1198
36	R0	{1.61457, 9.93364}	{1.67301, 19.016}	{{1.46788, 14.8506}, {1.89381, 20.}}	{9.07027, 91.43}	inf	112.	389.997	{295.824, 532.972}	248.212	0.530319	newly	110.	159.89	{105., 226.}	47.0636	0.154199	Reff	1.56904	1.53246	{1.37522, 1.72356}	8.14987	ar	6338.	7387.46	{6399., 8429.}	17.0164
37	R0	{1.47831, 7.08139}	{1.76874, 17.4198}	{{1.41009, 13.0427}, {2.20151, 20.}}	{21.5478, 145.994}	inf	63.	275.435	{159.645, 404.21}	337.199	0.612302	newly	82.	129.64	{70., 200.}	64.4634	0.195739	Reff	1.45954	1.66494	{1.32586, 2.11037}	18.8619	ar	5530.	7355.11	{5805., 8929.}	33.1839
38	R0	{1.73445, 9.8858}	{1.73758, 17.2799}	{{1.3746, 11.987}, {2.16805, 20.}}	{15.1473, 75.0083}	inf	67.	271.917	{151.853, 427.945}	305.846	0.575752	newly	71.	129.72	{73., 200.}	84.5352	0.232623	Reff	1.70548	1.6419	{1.29812, 2.05956}	14.2661	ar	6842.	7245.79	{5411., 8963.}	16.6494
39	R0	{1.66621, 11.4824}	{1.66743, 19.0032}	{{1.43302, 15.9352}, {2.03372, 20.}}	{10.5193, 65.4984}	inf	179.	484.049	{326.683, 648.535}	170.418	0.416696	newly	134.	194.63	{131., 261.}	47.0224	0.154999	Reff	1.58806	1.48841	{1.31307, 1.69681}	9.18306	ar	6473.	7378.86	{6193., 8516.}	15.7262
40	R0	{1.60694, 9.95741}	{1.59655, 19.0722}	{{1.44056, 18.1499}, {1.84282, 20.}}	{8.612, 91.5378}	inf	141.	416.191	{316.877, 553.978}	195.171	0.457853	newly	132.	167.28	{121., 232.}	30.1818	0.103318	Reff	1.54957	1.45396	{1.34325, 1.66786}	9.57488	ar	6625.	7044.12	{6152., 8289.}	9.89947
41	R0	{1.55128, 4.71911}	{2.00196, 13.4846}	{{1.1546, 6.93514}, {2.86001, 18.8819}}	{38.4068, 187.901}	inf	6.	148.98	{8.16113, 318.071}	2388.07	1.15252	newly	17.	103.32	{7., 206.}	526.941	0.696544	Reff	1.54988	1.90265	{1.14204, 2.64568}	33.9518	ar	6141.	6991.75	{2558., 9257.}	36.3467
42	R0	{1.60063, 6.81118}	{1.60152, 19.4895}	{{1.51326, 18.5842}, {1.72823, 20.}}	{4.02815, 186.14}	inf	35.	467.37	{390.348, 543.496}	1235.34	1.12109	newly	65.	181.21	{143., 210.}	178.785	0.438626	Reff	1.58799	1.45264	{1.36376, 1.57839}	8.93298	ar	6528.	7352.15	{6816., 7928.}	12.6999
43	R0	{1.56986, 5.44652}	{2.08123, 14.6136}	{{1.36126, 9.00474}, {2.98152, 20.}}	{38.7124, 168.804}	inf	8.	188.183	{68.3761, 359.131}	2252.28	1.28604	newly	23.	129.38	{33., 254.}	464.522	0.645333	Reff	1.56751	1.99373	{1.29077, 2.81035}	35.4271	ar	6312.	7707.92	{5172., 9569.}	31.7104
44	R0	{1.47523, 8.07785}	{1.62395, 18.8153}	{{1.43599, 14.4183}, {1.85894, 20.}}	{11.2597, 132.925}	inf	101.	372.327	{272.054, 529.313}	268.64	0.552838	newly	100.	153.35	{103., 220.}	53.93	0.171613	Reff	1.43968	1.49589	{1.35118, 1.68876}	7.88936	ar	5692.	7127.06	{6156., 8265.}	25.6005
45	R0	{1.6417, 7.69429}	{1.80495, 17.2821}	{{1.38664, 11.776}, {2.49504, 20.}}	{17.5976, 124.609}	inf	45.	283.406	{151.238, 481.481}	529.792	0.7586	newly	60.	136.15	{68., 225.}	128.017	0.314543	Reff	1.62545	1.70047	{1.3257, 2.28454}	15.7332	ar	6735.	7425.48	{5433., 9295.}	17.6659
46	R0	{1.38865, 5.83473}	{1.73229, 17.8244}	{{1.37034, 12.5676}, {2.1946, 20.}}	{25.4063, 205.489}	inf	64.	325.913	{185.154, 501.505}	409.239	0.666324	newly	101.	145.29	{81., 250.}	53.4752	0.162676	Reff	1.35977	1.60042	{1.29365, 1.90053}	19.7844	ar	5233.	7191.8	{5650., 8835.}	37.9473
47	R0	{1.45289, 6.87903}	{1.8463, 17.3292}	{{1.40481, 12.0502}, {2.57474, 20.}}	{28.7888, 151.914}	inf	78.	282.391	{156.021, 445.565}	262.04	0.522999	newly	104.	140.49	{66., 244.}	56.2981	0.195814	Reff	1.42441	1.75406	{1.32171, 2.40357}	25.9404	ar	5379.	7605.38	{5706., 9366.}	41.9814
48	R0	{1.69416, 9.4831}	{1.65596, 19.1176}	{{1.44951, 17.5196}, {1.87276, 20.}}	{9.76248, 101.597}	inf	47.	384.36	{278.582, 522.736}	717.786	0.899088	newly	41.	154.63	{105., 232.}	277.146	0.558591	Reff	1.67264	1.52707	{1.36191, 1.73949}	10.7758	ar	6925.	7359.47	{6448., 8335.}	10.2526
49	R0	{1.47843, 7.16286}	{1.73922, 17.9983}	{{1.40371, 13.8447}, {2.10809, 20.}}	{19.8895, 151.272}	inf	80.	286.902	{176.137, 435.498}	258.627	0.526208	newly	81.	126.96	{73., 191.}	61.7531	0.187854	Reff	1.44664	1.6351	{1.30594, 1.93778}	17.0524	ar	5449.	7308.99	{5577., 8618.}	35.388
50	R0	{1.68843, 5.72427}	{1.96636, 13.5989}	{{1.15409, 6.93514}, {2.86095, 18.8819}}	{30.9401, 142.119}	inf	14.	155.218	{10.489, 318.363}	1021.43	0.884796	newly	26.	116.54	{9., 207.}	371.769	0.59612	Reff	1.68522	1.86693	{1.12509, 2.71024}	28.093	ar	6800.	6833.71	{2426., 9275.}	28.8254
